# The effects on public health of climate change adaptation responses: a systematic review of evidence from low- and middle-income countries

**DOI:** 10.1088/1748-9326/ac092c

**Published:** 2021-07-13

**Authors:** Pauline F D Scheelbeek, Alan D Dangour, Stephanie Jarmul, Grace Turner, Anne J Sietsma, Jan C Minx, Max Callaghan, Idowu Ajibade, Stephanie E Austin, Robbert Biesbroek, Kathryn J Bowen, Tara Chen, Katy Davis, Tim Ensor, James D Ford, Eranga K Galappaththi, Elphin T Joe, Issah J Musah-Surugu, Gabriela Nagle Alverio, Patricia Nayna Schwerdtle, Pratik Pokharel, Eunice A Salubi, Giulia Scarpa, Alcade C Segnon, Mariella Siña, Sienna Templeman, Jiren Xu, Carol Zavaleta-Cortijo, Lea Berrang-Ford

**Affiliations:** 1 Centre on Climate Change and Planetary Health, London School of Hygiene and Tropical Medicine, London, United Kingdom; 2 Department of Population Health, London School of Hygiene and Tropical Medicine, London, United Kingdom; 3 Department of Public Health, Environment and Society, London School of Hygiene and Tropical Medicine, London, United Kingdom; 4 University of Leeds, Priestley International Centre for Climate, Leeds, United Kingdom; 5 Mercator Research Institute on Global Commons and Climate Change, Berlin, Germany; 6 Department of Geography, Portland State University, Portland, OR, United States of America; 7 Ostbayerische Technische Hochschule Amberg-Weiden, Bayern, Germany; 8 Wageningen University and Research Centre, Wageningen, The Netherlands; 9 Melbourne School of Population and Global Health, University of Melbourne, Melbourne, Australia; 10 Institute for Advanced Sustainability Studies, Potsdam, Germany; 11 E-Da Hospital, Kaohsiung City, Taiwan; 12 Department of Fisheries and Oceans Canada, Winnipeg, Manitoba, Canada; 13 Department of Geography, Virginia Tech, Blacksburg, VA, United States of America; 14 World Resources Institute, New Delh, India; 15 United Nations University Bonn, Bonn, Germany; 16 Nicholas School of the Environment, Sanford School of Public Policy, Duke University, Durham, NC, United States of America; 17 Heidelberg University, Heidelberg, Baden-Württemberg, Germany; 18 The University of Sheffield, Sheffield, United Kingdom; 19 University of Waterloo, Waterloo, Ontario, Canada; 20 CGIAR Research Program on Climate Change, Agriculture and Food Security, International Crops Research Institute for the Semi-Arid Tropics, Bamako, Mali; 21 Faculty of Agronomic Sciences, University of Abomey-Calavi, Cotonou, Benin; 22 Universidad Peruana Cayetano Heredia, Lima, Peru; 23 Columbia University, New York, NY, United States of America; 24 University of Glasgow, Glasgow, United Kingdom

**Keywords:** climate change adaptation, climate change adaptation response, public health, systematic review, data synthesis, low- and middle-income countries

## Abstract

Climate change adaptation responses are being developed and delivered in many parts of the world in the absence of detailed knowledge of their effects on public health. Here we present the results of a systematic review of peer-reviewed literature reporting the effects on health of climate change adaptation responses in low- and middle-income countries (LMICs). The review used the ‘Global Adaptation Mapping Initiative’ database (comprising 1682 publications related to climate change adaptation responses) that was constructed through systematic literature searches in Scopus, Web of Science and Google Scholar (2013–2020). For this study, further screening was performed to identify studies from LMICs reporting the effects on human health of climate change adaptation responses. Studies were categorised by study design and data were extracted on geographic region, population under investigation, type of adaptation response and reported health effects. The review identified 99 studies (1117 reported outcomes), reporting evidence from 66 LMICs. Only two studies were *ex ante* formal evaluations of climate change adaptation responses. Papers reported adaptation responses related to flooding, rainfall, drought and extreme heat, predominantly through behaviour change, and infrastructural and technological improvements. Reported (direct and intermediate) health outcomes included reduction in infectious disease incidence, improved access to water/sanitation and improved food security. All-cause mortality was rarely reported, and no papers were identified reporting on maternal and child health. Reported maladaptations were predominantly related to widening of inequalities and unforeseen co-harms. Reporting and publication-bias seems likely with only 3.5% of all 1117 health outcomes reported to be negative. Our review identified some evidence that climate change adaptation responses may have benefits for human health but the overall paucity of evidence is concerning and represents a major missed opportunity for learning. There is an urgent need for greater focus on the funding, design, evaluation and standardised reporting of the effects on health of climate change adaptation responses to enable evidence-based policy action.

## 1. Background and justification

Evidence on the links between climate change and human health is accumulating rapidly [1, 2] and the various direct and indirect pathways through which climate change affects health are increasingly being mapped and measured [2–4]. Much available literature highlights that the effects on public health from climate change are predominantly and most severely experienced among poor populations in low- and middle-income countries (LMICs) [5, 6], however the evidence base in these settings is also the most scant [7]. For effective evidence-based policy action in LMICs, there is an urgent need to strengthen the evidence base on the effect on health of climate change adaptation responses [8].

A number of global and regional analyses and modelling studies are providing evidence on the potential efficacy of various adaptation responses [9], including adaptations in agriculture [10–12], built environment [13], and/or behaviour change [14]. However, their effectiveness, effects on human health, as well as the context-specific enablers and barriers for uptake are less well-known [15–17]. There are several possible explanations for this lack of evidence. First, time and financial constraints do not always allow for a comprehensive evaluation of climate change adaptation responses and their health effects [18]. Second, the nature of the climate hazard (that can lead to ad hoc cascading, complex and unpredictable responses) frequently limits possibilities for formal evaluation. Third, some evaluations only focus on a restricted part of the pathway between climate change and health, such as the effect of drought-tolerant crops on food availability or the effect of increased green space on days with extreme heat. Fourth, some evaluations only focus on measures of uptake but do not evaluate the short or long-term effects on population health. And finally, collating the evidence base is challenging because of reporting heterogeneity and lack of widely recognised and applied reporting standards [19].

Comprehensive, (potentially continuous), and up-to-date reviews of evidence of the effects of adaptation responses on health from (local) empirical evaluations are essential for several reasons. First, access to state-of-the-art synthesised evidence may enhance and streamline the development of climate change adaptation responses that support public health. Furthermore, evaluations of case studies—conducted in a diverse range of contexts—would be a crucial part of this: it would allow identification of ‘best practices’, contextual determinants of success, co-benefits for other sectors, synergies with climate change mitigation efforts, and would enable learning from previous challenges or successes.

The current study seeks to contribute to a growing literature aiming to identify, document, and evaluate the impacts of adaptation responses, as well as assess progress in climate change adaptation approaches [20–22]. In particular, we have sought to improve how we conceptualise and assess the effects on health of adaptation options. Existing work in this area is largely focussed on high-income nations [2, 23, 24]; in contrast, our systematic evidence synthesis of peer-reviewed studies focusses on the reported effects on health of climate change adaptation responses in LMICs. We discuss challenges in systematic reviews and evidence synthesis of literature on climate change adaptation and health, including issues around evaluation quality and reporting. Finally, we propose guidance for improved reporting of climate change adaptation and health evidence, that would enable faster and more efficient evidence synthesis for LMIC contexts.

## 2. Methods

This review follows the preferred reporting items for systematic reviews and meta-analyses (PRISMA) guidelines [25] and follows the ROSES flow-chart [26]. We summarised the available published evidence on the effect on health of climate change adaptation responses in LMICs.

We used an existing database, initially constructed as part of the global adaptation mapping initiative (GAMI) [27] that mapped and reviewed the published literature on climate change adaptation responses in human systems. The search methods of the GAMI database are made available in detail elsewhere [27–30]. In short, systematic literature searches were performed in Scopus, Web of Science Core Collection and Google Scholar for the years 2013–2020. The search strategy used a combination of terms related to climate change, global warming, adaptation and resilience to identify studies that reported on climate change adaptation. The GAMI database retrieved 48 816 publications and following screening these were narrowed down to a database of 1682 publications relevant to human adaptation responses to climate change. All further screening and coding described below was performed manually by the authors of the current study.

For the current project, titles and abstract of all papers in this database of 1682 publications were screened (by PS, SJ, GT and/or LBF) to identify studies that specifically reported the effects on human health of climate change adaptation responses. We framed our review using a SPIDER-approach: the sample (S) was all LMICs; the phenomena of interest (PI) was the effect on health of climate change, climate variability and weather adaptation responses (hereafter referred to as ‘climate change adaptation responses’) and includes planned adaptations as well as unplanned rapid responses such as disaster response activities; we included all empirical study designs (D) as well as empirically-driven modelling studies and reviews; our aim was to evaluate (E) the effect on health of climate change adaptation responses; and as per the GAMI protocol, we included both qualitative and quantitative research (R). Our time period was 2013–2020 to include studies published since the release of the Intergovernmental Panel on Climate Change (IPCC) 5th Assessment Report (AR5 [1]).

Studies were included for this review if they met all of the following four criteria: (a) the study was performed in a LMIC as per Word Bank definition [31]; (b) the study evaluated the impact of a climate change adaptation responses; (c) the study clearly stated the climate hazard(s) addressed by the adaptation; and (d) the study reported quantitative or qualitative data (including more peripheral comments or assertions) on any health outcome (such as mortality or disease prevalence) or on any intermediate health outcome (such as food security, or indicators of water, sanitation and hygiene status) at individual and/or community-level.

All included studies were assessed in duplicate (by PS, SJ, GT, ADD and/or LBF) for robustness and relevance and assigned a study design category (1–4), defined as follows:

• Category 1: studies that report formal *ex ante* planned and designed evaluations of adaptation responses (with a counterfactual), and feature a baseline and follow-up measurement (or comparison between two areas) of specified health outcomes.
• Category 2: studies that do not report formal *ex ante* evaluations but assess the impact of adaptation responses on specified health outcomes using a ‘comparison’ area (typically that did not adapt) or by using previously collected baseline data (‘before and after’ design).
• Category 3: studies that report specified health outcomes with no counterfactual.
• Category 4: studies that provide qualitative comments or assertions on the effects of climate change adaptation responses on health with little supporting evidence.

Relevant data extracted from included papers included the type of climate hazard evaluated (e.g. drought, flood, precipitation variability), the adaptation response category (e.g. technological, behavioural, health system); the reported health outcomes, and the reported effects on health and their units of measurement. Information and analyses related to costs were beyond the scope of this study. *Ex ante* categorisations of climate hazards, adaptation responses and health outcomes (*ex ante* defined by authors of the current study) were complemented by emerging categories from the identified literature. We extracted contextual factors including year of study, geographic region (e.g. continent, urban vs rural, coastal vs inland) and population under investigation (e.g. general population, farmers, children) and extracted information on maladaptation when reported by the study authors. The extracted data were collated into a database, and categorised by climate hazard, adaptation response, and health outcome.

We analysed and mapped the geographic distribution of studies, produced a heat map of adaptation responses and health outcomes, and included a narrative synthesis—including all of the 99 identified papers—by four themes: geographic location; vulnerable target population; climate hazard and type of adaptation response; and health outcome. Given the heterogeneity of the available data, limited formal quantitative analysis was possible. Summary statistics were calculated for health outcomes with a minimum of five studies reporting quantitative results: in this study this was only possible for food security outcomes). A searchable spreadsheet of included papers is available through the LSHTM data repository Data Compass (datacompass.lshtm.acuk), and included studies are listed in the supplementary files (available online at stacks.iop.org/ERL/16/073001/mmedia).

## 3. Results

### 3.1. Identified studies

**Figure 1.**
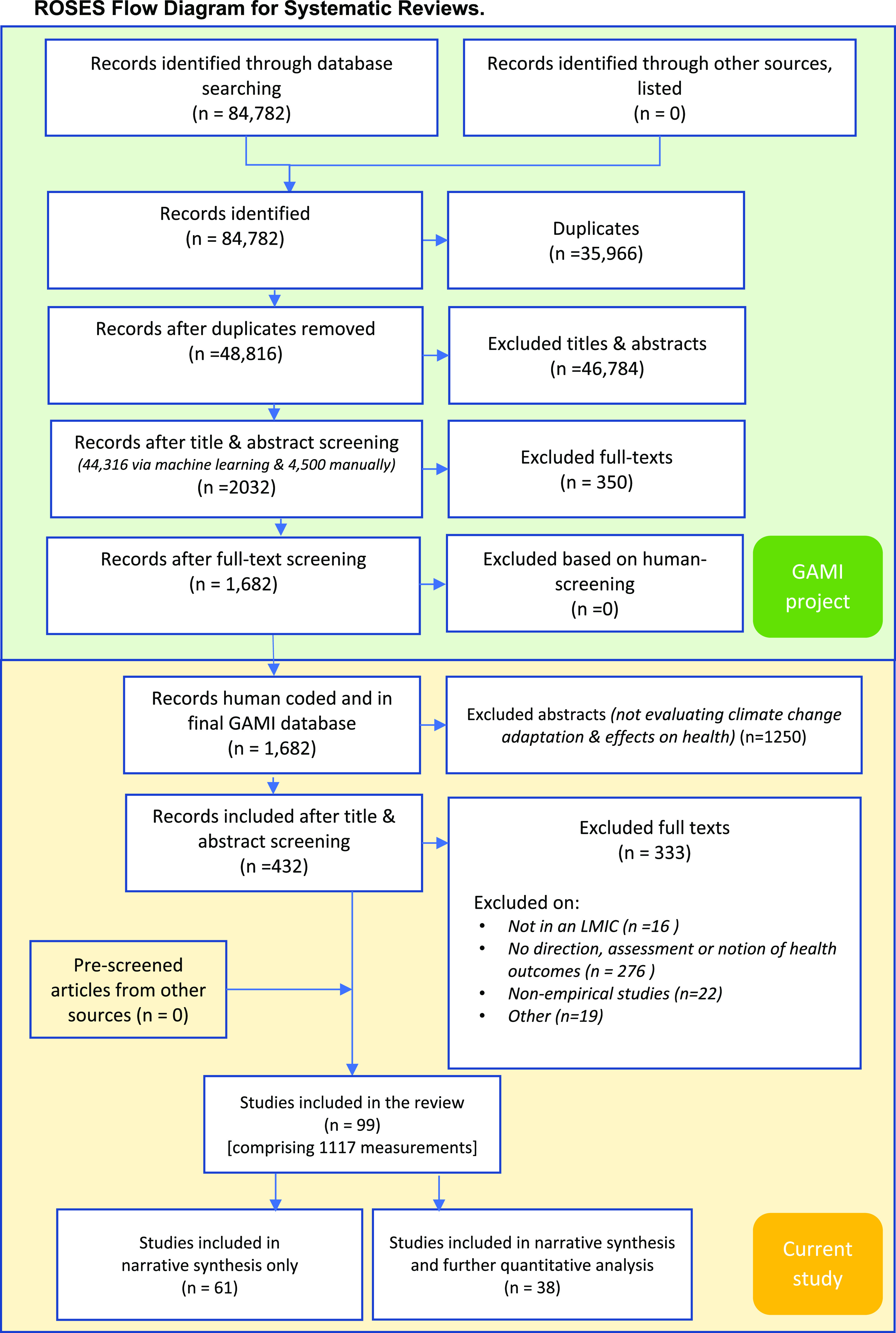
ROSES diagram of literature screening. Screening steps in the green coloured area were part of the GAMI, and the screening steps in the yellow area were for the current study.

We included 99 studies that were conducted in 66 different LMICs and reported 1117 unique measurements or mentions of the effect on human health or intermediate health outcomes of adaptation responses (figure 1). Thirty-eight studies reported quantitative health outcomes and were available for both quantitative analysis and narrative synthesis. Sixty-one studies provided only qualitative data and are reported here through narrative synthesis.

We identified seven climate hazard categories and nine climate change response categories (table 1). Adaptation response categories were further grouped into three aggregate categories (improving community resilience, policy, governance and finance, and disaster risk reduction). The studies reported on five health outcomes and three intermediate health outcomes with the remainder of studies grouped into an ‘other health outcomes’ category.

**Table 1.** Numbers of studies included in systematic review by categories of climate hazard (A), adaptation response (B) and health outcome (C).

**(A) Climate hazard**		
Description of hazard		*n*
Extreme precipitation and inland flooding		46
Increased frequency and/or intensity of heat		32
Precipitation variability		43
Drought		59
Rising ocean temperature and ocean acidification		4
Sea level rise and coastal flooding		24
Combined/general climate change impacts		57
**(B) Adaptation response**		
**Description of adaptation response**	**Adaptation response aggregate**	***n***
Behaviour change	Improving community resilience	50
Capacity building		8
Communication, information and raising awareness		13
Technological advancement	Disaster risk reduction	32
Green infrastructure (including climate smart agriculture)		37
Physical infrastructure improvements		31
Early warning systems		11
(Micro)-financing	Policy, governance and finance	20
Adaptation through policy options		14
**(C) Health outcome**		
**Description of health outcome**	**Health outcome aggregate**	***n***
All-cause mortality	Health outcomes	9
Infectious disease		17
Non-communicable disease		2
Mental health		5
Maternal and child health		0
Food security indicators	Intermediate health outcomes	46
Water, sanitation and hygiene (WASH) indicators		16
Health system indicators		5
Other health outcomes	Other	13

**Figure 2.**
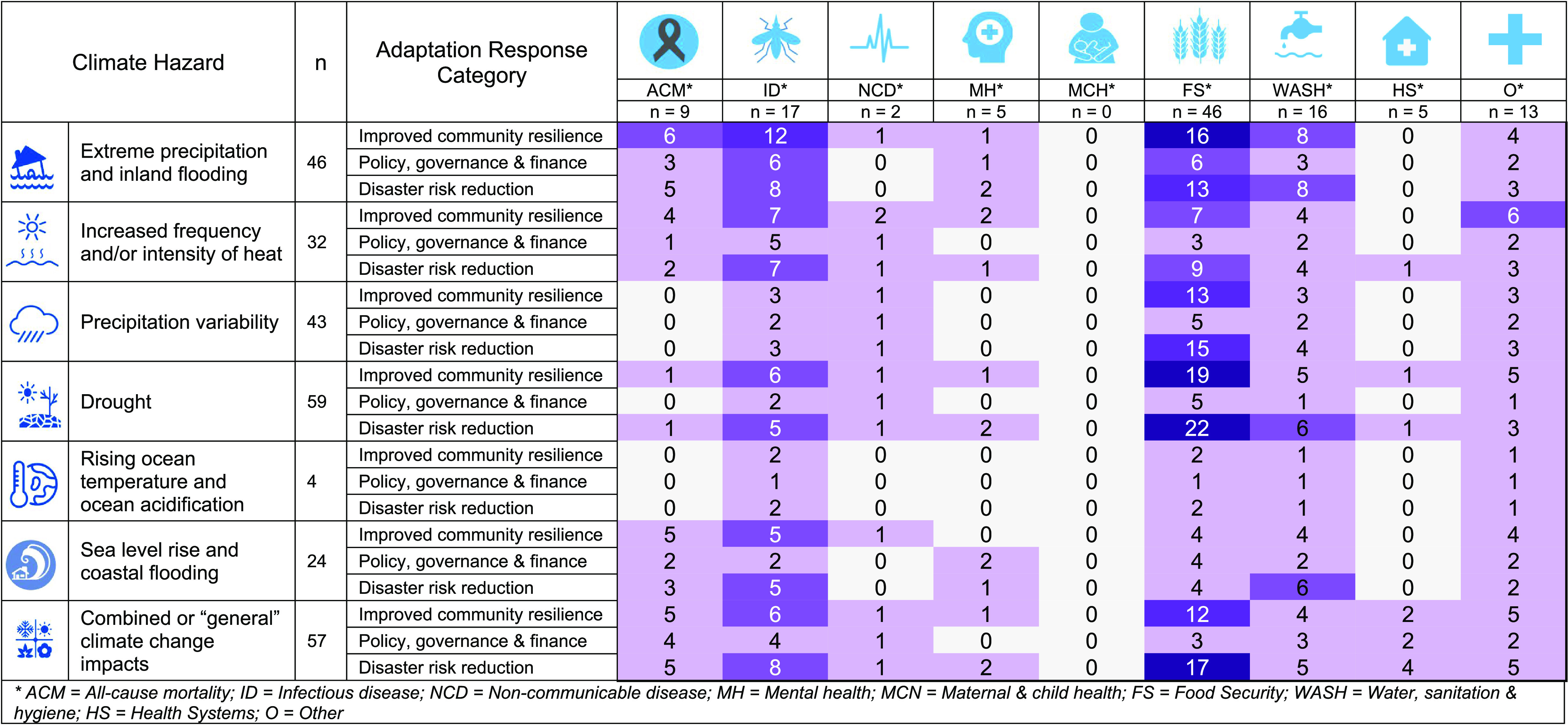
Heat map of the various climate drivers and health outcomes—by adaptation category: improving community resilience; policy, governance and finance; and disaster risk reduction. Several studies assessed the impact of multiple climate hazards, adaptation categories and/or health outcomes.

**Figure 3.**
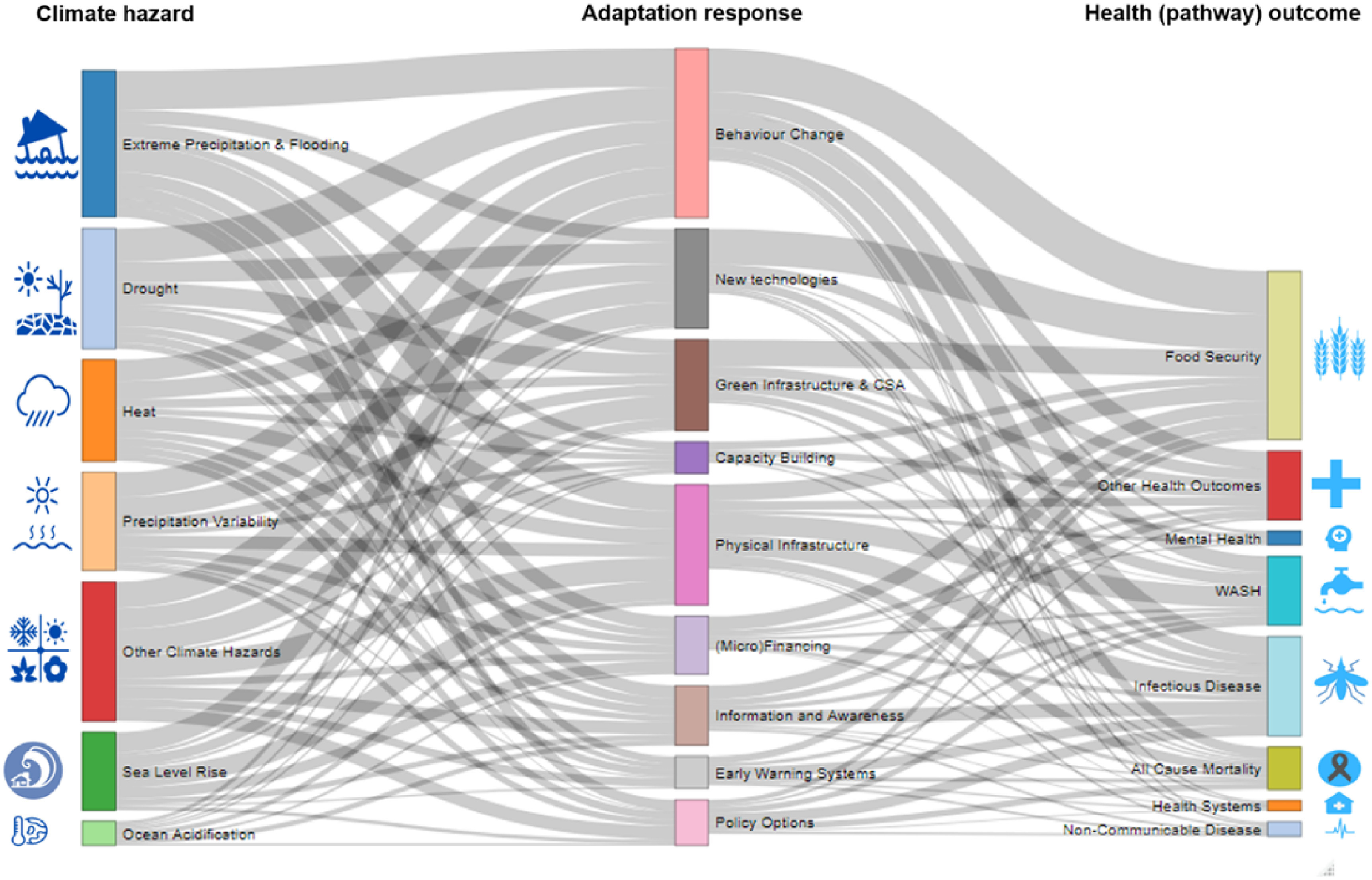
Sankey diagram of climate hazard, adaptation responses and health outcomes. Diagram represents all individual study outcomes and data points (*n* = 1117) reported in the studies included in this review. (CSA = climate smart agriculture).

The most common reported climate hazards were drought (59 studies), extreme precipitation (46 studies), increased heat (32 studies), and precipitation variability (43 studies). The most common adaptation responses were related to behaviour change (50 studies), green infrastructure (including certain climate smart agriculture (CSA) activities (37 studies)), new technologies (32 studies), and other infrastructural improvements (31 studies). The most commonly reported health outcomes were related to infectious diseases (17 studies). No study reported on the *ex ante* defined category of maternal and child health outcomes. The evidence base was largest for studies evaluating the effect of adaptation responses on intermediate health outcomes, especially outcomes related to food security (46 studies) and water, sanitation and hygiene (WASH; 16 studies) (table 1). Studies typically reported multiple combinations of climate hazards, health outcomes, and adaptation responses, and a total of 1117 data points were extracted. The size of the evidence base, for all possible combinations of climate hazards, adaptation responses and health outcomes, is depicted by the number of studies in figure 2 and number of reported outcomes (including multiple outcomes reported by individual studies) in figure 3. The identified exposure—adaptation—health outcome pathways evaluated in each study are listed in the supplementary materials along with disaggregated data on the specific health outcomes evaluated in each study.

### 3.2. Geographical distribution of evidence

**Figure 4.**
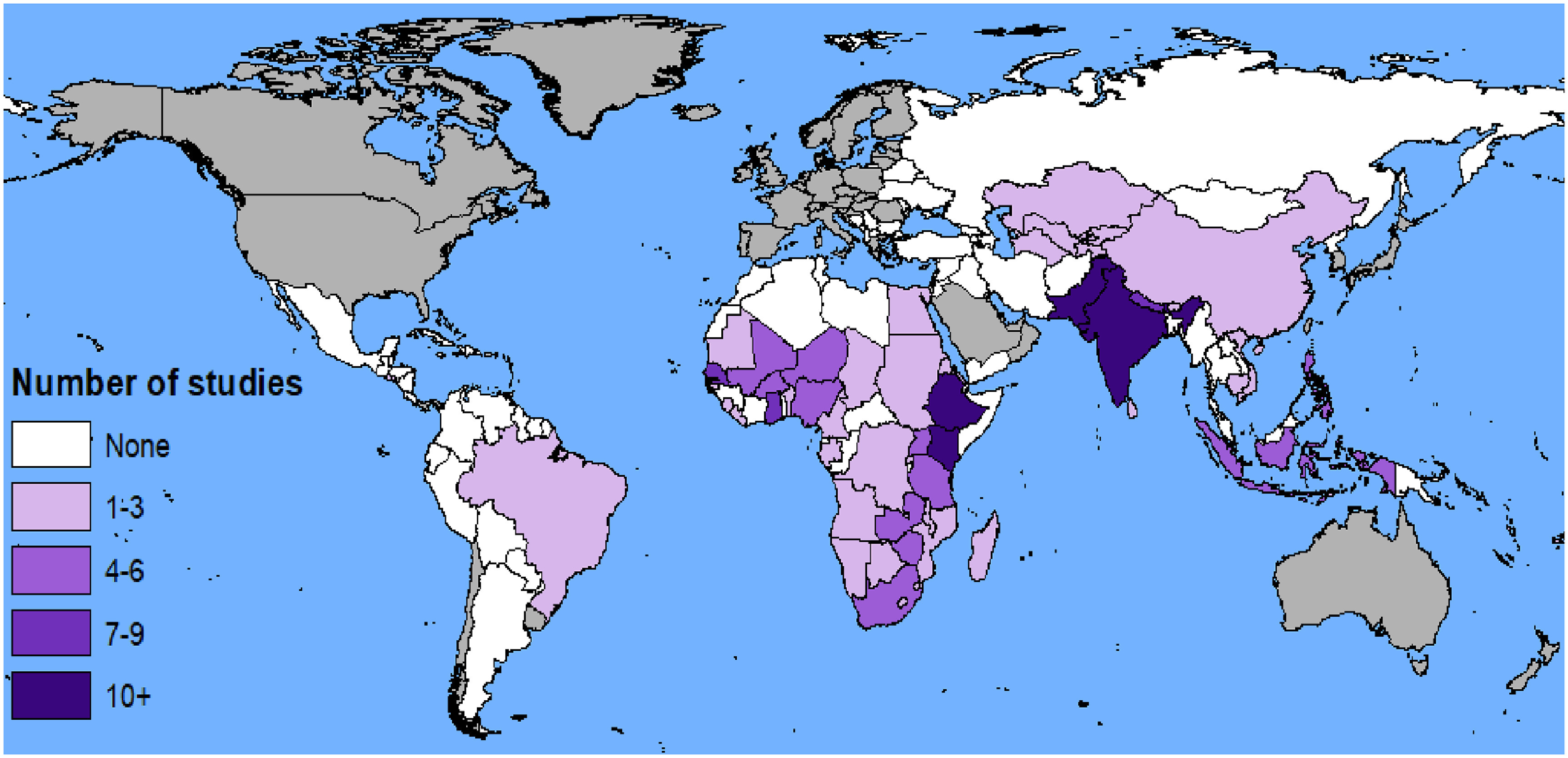
Global distribution of included studies and their relative abundance. (The review did not cover high income countries [in grey]).

Among the identified studies in LMIC settings, the majority were reporting on studies conducted in Asia (49 studies, with India (*n* = 11) and Bangladesh (*n* = 14) as most frequently reported countries) and Africa (38 studies, with Ethiopia (*n* = 9) as most frequently reported country). Seven studies were global studies, and one study assessed health outcomes in 57 small island states (figure 4).

### 3.3. Study designs

**Figure 5.**
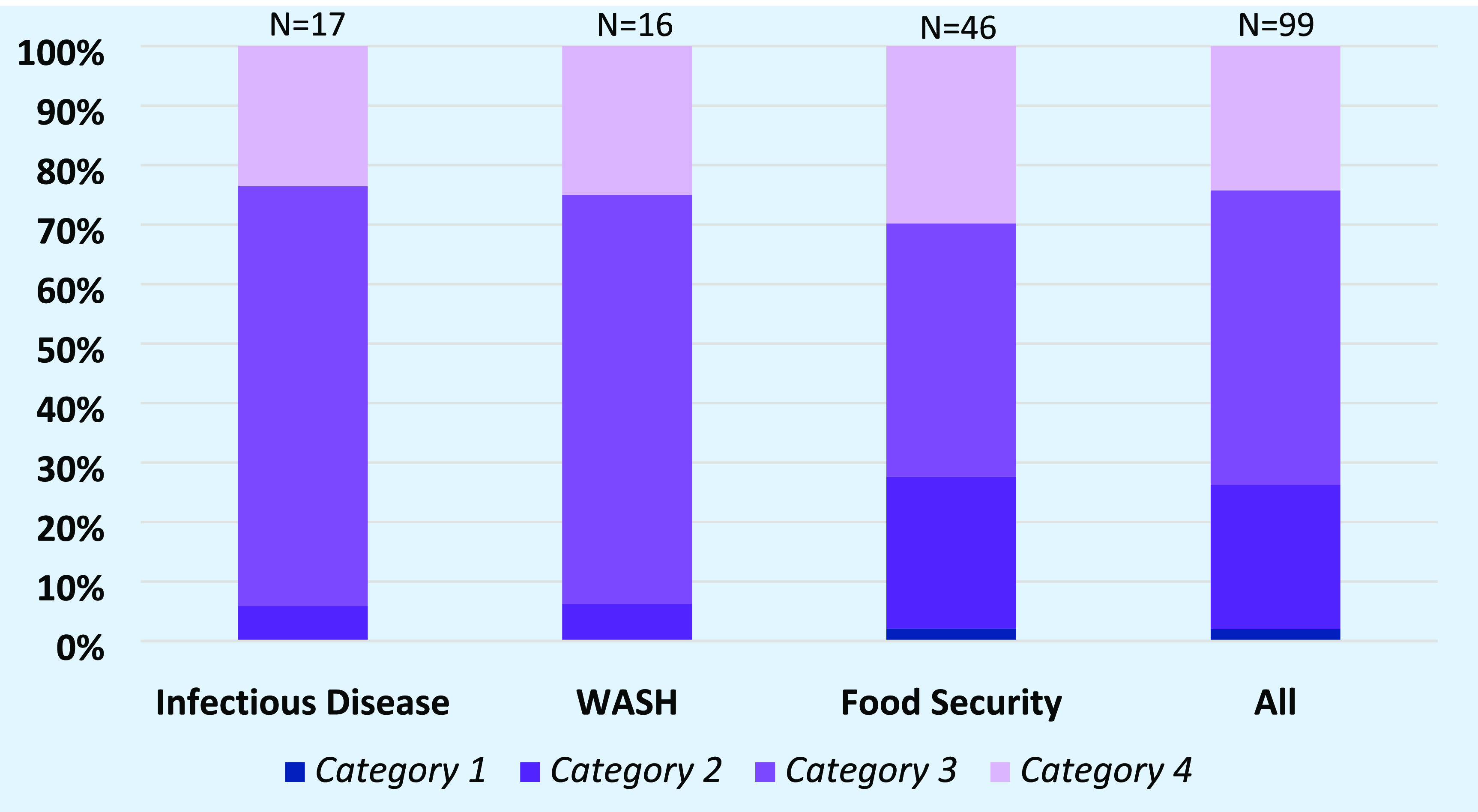
Percentage of studies in each study design category for studies reporting on infectious disease, WASH and food security, as well as an all studies combined.

We identified two studies with a ‘category 1’ study design, and a relatively small number (*n* = 24) of studies assessed as category 2. The majority of studies were assessed as ‘category 3’ and ‘category 4’ study designs (49 and 24 studies, respectively). Studies reporting on food security showed the highest proportion of ‘category 1’ and ‘category 2’ study, and these typically reported more contextual details as well as more comprehensive quantitative analysis of the effects on health of the evaluated climate change adaptation responses (figure 5).

### 3.4. Evidence synthesis of selected health outcomes

Given the distribution of the evidence, we focus our evidence synthesis on the three most commonly reported outcomes namely, infectious disease, WASH and food security.

#### 3.4.1. Infectious disease

Seventeen studies reported on the effects of adaptation responses on measures of infectious disease. **Geography:** Studies reported on Asia (5), Africa (5) and Oceania (1), whilst six studies reported on multiple countries and/or reported outcomes in the global context. Specific geographies of studies were mixed as some were in coastal areas (8), inland (6), urban (8), and rural (5). **Study populations:** Five studies reported on specific populations; children (1), environmental health practitioners (1), low-income groups (1) and farmers (2). **Study design and category:** The majority of studies were cross-sectional studies (9), three studies were qualitative, one study was longitudinal and five were reviews. More than 90% of the included evidence base derives from studies in category 3 or 4 (figure 5).

**Climate hazards:** The most commonly reported climate exposure was extreme precipitation and inland flooding (12), droughts (7), increased heat (6) and coastal flooding (5). **Adaptation responses:** Commonly reported adaptation responses were related to practice or behaviour change (12), physical infrastructure improvements (10) and information, communication or raising awareness (7). They included adaptation responses such as migration, safe water storage, domestication of animals as alternative food source and securing livestock, enhanced infectious disease control programmes, breeding site inspection and vector awareness campaigns. **Pathways to health:** The primary reported pathways were household and community-level adaptations that reduced the impacts of flood and thereby led to a reduction in the prevalence of vector borne diseases (dengue and malaria). Other pathways included the link between water conservation and water-borne and water-washed diseases including diarrhoea and cholera. One study reported on dengue incidence.

**Qualitative and quantitative results:** Thirteen studies (that fell in study design category 1, 2 or 3) reported data on the effect on infectious disease of climate change adaptation responses; eight reported a beneficial response, two reported a negative response and three reported mixed responses. Four studies reported quantitative information on infectious disease incidence in 38 individual outcome measurements. Reported changes in incidence of infectious disease resulting from the adaptation activities across all sub-groups ranged from −18% to +6%. The largest positive effects were reported to result from physical infrastructure change, whilst behaviour change and awareness activities commonly showed no effects.

#### 3.4.2. WASH indicators

Sixteen studies reported on the effects of adaptation responses on WASH indicators. **Geography:** Studies reported on Asia (7), Oceania (2), Africa (1), Central and South America (1) or reported on multiple countries and/or the global context (5). Eight studies were conducted in coastal settings and eight in rural settings, whilst six focussed on urban areas, six on rural areas, and one study focussed on both urban and rural settings. **Study populations:** Twelve studies assessed the effect of adaptation responses on health of the general population, two studies reported on farmers, one on environmental health practitioners, and one on low-income households or communities. **Study design and category:** Most included studies used a cross-sectional design (9) or were reviews (6): there was one expert elicitation. More than 90% of the included evidence base derives from studies in category 3 or 4 (figure 5).

**Climate hazards:** The most commonly reported climate exposure was extreme precipitation and inland flooding (9), droughts (7), and coastal flooding (5). **Adaptation responses:** The most commonly evaluated adaptation responses were related to behaviour change approaches (11), physical infrastructure improvements (13), and ecosystem responses (7), though most studies (12) evaluated a combination of adaptation strategies. **Pathways to health**: Adaptation responses included small dams and flood barriers to reduce inundations in the home, and rainwater storage infrastructure. Several studies evaluated the impact of storing water for irrigation on food security.

**Qualitative and quantitative results:** Twelve studies (that fell in study design category 1, 2 or 3) reported on the effectiveness of climate response activities; eight reported a beneficial response, and four reported mixed responses. Adaptation responses that aimed to reduce home flooding (*n* = 4) all found a significant reduction in gastro-intestinal disease risk, skin infections and injuries. Two studies reported quantitative information on change of WASH indicators (as result of the adaptation response) in eight individual outcome measurements and reported changes in WASH indicators ranged from +1% to +47%.

### 3.5. Food security and malnutrition

Forty-six studies reported on the effects of adaptation responses on food security and malnutrition. **Geography:** Studies reported on Africa (28), Asia (11), Oceania (3) and on the global context (4). Twelve studies were conducted in coastal settings, of which five focussed on rural areas, and seven studies focussed on both urban and rural settings. 24 rural studies, and two urban-rural combination studies reported on non-coastal settings; two studies did not report on urbanicity**. Study populations:** Twenty studies assessed the effect in the general population and 20 reported on farmers, one study looked into male versus female led households. **Study design and category:** The majority of studies (35) used a cross-sectional study design, five studies were reviews, four studies were longitudinal and two studies were empirical modelling studies. Nearly 30% of the included evidence base derives from studies in category 1 or 2 (figure 5).

**Climate hazards:** The majority of studies reported responses droughts (29), precipitation variability (18) and extreme precipitation and inland flooding (18) while also a considerable number of studies focussed on more general climate change impacts (19). **Adaptation responses:** The most commonly evaluated adaptation responses were related to behaviour change approaches (20), technological advances (19), and ecosystem responses or green infrastructure, including CSA (17). Approximately half of the studies (24) evaluated a combination of adaptation strategies. **Pathways to health**: Reported adaptation responses fell into three categories: expanding agricultural produce to different/additional products (including tree planting); cultivation of climate resilient crop varieties or the application of climate resilient agricultural management strategies; and enhanced storage or preservation techniques of foodstuffs for consumption at times of reduced food supply. The disaggregated food security indicators that fall under each of these categories can be found in the supplementary table (supplementary files).

**Figure 6.**
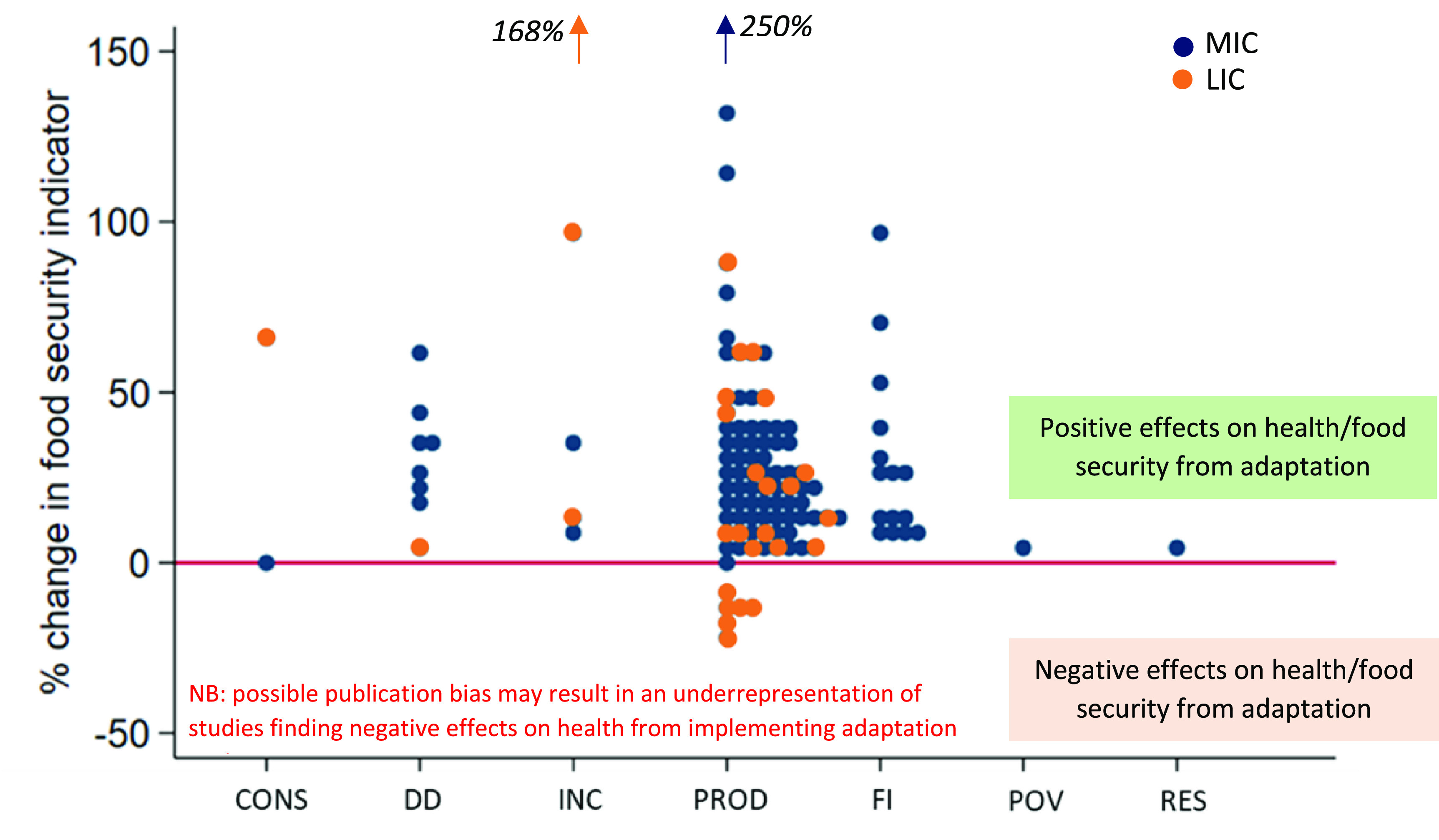
The effect on food security indicators of climate change adaptation strategies (expressed in per cent change compared to non-adaptors or baseline measurements). Indicators related to: CONS = consumption, DD = dietary diversity, INC = income, PROD = production, FI = food insecurity; POV = poverty, RES = resilience. MIC = middle income country; LIC = low income country.

Thirty-eight studies (all 33 studies in study design category 1, 2 and 3 and in addition five studies in ‘category 4’) reported the effectiveness of adaptation responses; 24 reported a beneficial response; two negative responses and 12 reported mixed responses. Eighteen studies reported quantitative information on change in food security indicators (as result of the adaptation response) in 117 individual outcome measurements. Reported changes in food security indicators from adaptation responses ranged from −18% to +133% with two outliers reporting on larger food security improvements. Reported improvements in production due to climate change adaptations were, on average, higher in middle-income countries (28.7% (95% CI 22.5–34.8)) than in low-income countries (17.9% (95% CI 6.03–29.8)) after removing outliers (figure 6).

The studies reported a large variety of food security outcomes, often evaluating multiple combinations of climate exposures, and therefore the results could not be pooled. The largest food security improvements were reported from projects that evaluated the impact of climate resilient crop varieties (often under the heading of CSA), and advancements in agricultural management.

#### 3.5.1. Other health outcomes

A small number of studies reported on the effect of climate change adaptation responses on a range of other health outcomes. Nine studies (of which six were from Asia) reported on all-cause mortality and evaluated a range of climate change response activities, including those aiming to reduce the impacts of heat, flood, air pollution and other climate disasters on population mortality. All studies reported that (various) adaptation responses reduced all-cause mortality, though three studies found negative health effects for some subgroup analyses. Five studies reported on adaptation responses and mental health, and two reported on non-communicable disease, with all studies reporting positive health effects resulting from the evaluated adaptation activities. Thirteen studies reported on other health outcomes, including respiratory illnesses and well-being.

### 3.6. Maladaptation

Nearly a 3rd (*n* = 31) of papers reported on maladaptations related to the evaluated climate change responses [32]. These maladaptations can be summarised in four themes. First, there are a number of papers describing maladaptation related to gender inequalities. For example, there were major differences reported in enablers and barriers to uptake of some adaptation responses between men and women. Increased and excessive workload on women related to the climate change adaptation response was mentioned frequently, whilst others (especially migration related responses) were reported to more danger to women and/or female headed households. Secondly, a number of studies report that improved resilience and positive effects on health at the individual level, did not necessarily translate into improvements of resilience of the system, especially when certain underlying systemic problems (e.g. education and size of the livestock herd) were not addressed. For that reason, several studies expressed their concerns over the longer-term benefits of the adaptation responses evaluated. A 3rd category related to unexpected or hidden co-harms including the perceived reduction of well-being related to infrastructural development, negative health and environmental consequences of self-driven diversification (for example coral mining) and stress and emotional pressure related to migration. Finally, a 4th category of reported maladaptations was related to pre-existing socio-economic disparities and inequalities and associated maladaptation of responses. This was frequently mentioned in adaptation strategies involving loans, whereby the poorest households particularly struggled to make their repayments. Some non-financial adaptation responses that required a relatively high investment costs were also reported to have limited uptake among the poorest households.

## 4. Discussion

### 4.1. Key findings

Our study has identified a disparate and limited evidence base on the effects on health of climate change adaptation responses in LMICs. There is a concerning lack of *ex ante* formal evaluations of climate change adaptation responses and our review found only 38 studies in total that reported quantitative data, precluding limited pooled analysis. Evaluation timelines were typically short with no studies reporting on longer term health outcomes (greater than 12 months). Furthermore, the methods and results of many included studies were frequently poorly reported. The majority of papers evaluated adaptation responses to extreme weather events: evaluation of adaptation options for gradual climate change were scarce, while evidence on these are also of pivotal importance to safeguard population health. We identified a very limited evidence base on major health indicators such as all-cause mortality, and no evidence on other important outcomes in LMICs including maternal and child health.

The largest evidence base reported on the effect of adaptation responses on infectious disease, and indicators of WASH and food security. There is limited evidence that some climate change adaptation responses improved WASH and food security outcomes and this may be important for future response planning. The preponderance of positive outcomes reported in included studies suggests that reporting and publication bias is likely, further limiting the opportunities to provide a robust evidence synthesis and draw valid conclusions. It is noteworthy that a wide spectrum of maladaptations were reported underlining the need to understand the contextual determinants of successful adaptation responses for improved population health.

### 4.2. Results in context: relevance for policy research and practice

There has been a growing recognition, over the past decade, that the effects of climate change adaptation responses on health need to be understood in more detail [33]. However, the evidence base, specifically in LMICs, and our knowledge on specific barriers and enabler for success, remains relatively small. In 2011, a systematic search of United Nations Framework Convention on Climate Change (UNFCCC) initiatives to identify evidence on tangible adaptations and their effects on health concluded that adaptation actions were largely at groundwork stage and that implications for health were absent in initiatives of any of the 38 contributing countries [21]. Furthermore, consideration for the special needs of vulnerable groups was broadly underdeveloped. Similar to the current study, they found that WASH and food security outcomes were priority themes. A 2014 report [22] evaluated 54 published studies related to national-level adaptation planning with a specific focus on infectious disease risks in OECD countries and identified a lack of consideration of the needs of vulnerable population groups as well as very limited evidence on local context-specific factors that could influence adaptation. The authors argue that a possible reason for evidence paucity could be linked to the lack of available funding and timelines for evaluation, and/or an evaluation component not being considered as a priority in climate change adaptation action. A more recent systematic review of grey literature on the effects on health of climate change adaptation in ten OECD countries suggested that governments primarily address infectious disease and heat-related risks posed by climate change [20]. Finally, a review of project reports from the 1st 5 years of implementation (2008–2013) of multinational health adaptation projects in ten LMICs identified that countries remain insufficiently prepared to prevent additional health burdens resulting from climate change [33].

Our study extends the current evidence base by including the most recent published literature and specifically focussing on LMICs. We conclude that evidence paucity remains a significant problem with major knowledge gaps and limitations in socio-economic, demographic and vulnerability aspects. The clear and concerning lack of evidence, notably the lack of measured quantitative information, poses major challenges for evidence-based decision-making regarding climate change adaptation options.

### 4.3. Priorities for the decade to come

We propose a number of priorities for study planning, reporting and publishing that—if adopted over the coming years—would be major leverage points for enabling high-quality, comprehensive and valid data synthesis through systematic reviews of the literature.

**Study planning**—A major reason for data paucity may relate to research capacity and preparedness for data collection. As many countries in Sub-Saharan Africa and South and South-East Asia are disproportionately affected by climate change, improving the research capacity in these countries to conduct climate change adaptation evaluations, should be prioritised. This includes both preparation (e.g. set-up and protocols for data collection) prior to the occurrence of climate hazards (including disasters) as well as tailor-made research training that incorporates a careful consideration of contextual factors or hazards that could affect climate change adaptation evaluations. Interactive webinars and training materials on accessible platforms could improve access to curricula on research and evaluation training and encourage continuous improvement and adaptation of the training materials by the climate change adaptation community.

**Funding**—Formal evaluations are resource-intensive and a lack of available funding will directly limit the conduct of high-quality evaluations. We urge research funders to acknowledge the crucial importance of evidence on the effects on health of climate change adaptation for evidence-based decision making over the coming years. This would also enable the evaluation of longer-term health impacts of adaptation options and allow more in-depth data analysis linked to existing (routinely collected) health data.

**Study protocol guidance and reporting templates**—We strongly advocate for the development of clear and open access data collection and reporting templates for climate change adaptation evaluations—and in particular those that that aim to measure effects on health. The use of experimental designs, such as community based randomised controlled (or step-wedge) trials should be actively encouraged. Reporting should be standardised to capture a minimum set of data, including (but not limited to) study context, full details and quantification of health outcome, equity indicators, maladaptation, and financial and time costs of the adaptations.

**Context and equality**—Further research should investigate not only the types of adaptation measures adopted, but quantify how effective they are in achieving the goals formulated, among different groups in society, and how feasible implementation of the options are in various context.

**Tackling reporting and publication bias**—We strongly encourage journals editors, medical associations, funders, researchers, and NGO representatives to prioritise identifying solutions for reporting and publication bias in this literature. An international repository of climate adaptation strategies and reports that is readily accessible may support the dissemination of key findings, lessons learned, and best practices.

### 4.4. The role of machine learning

Evidence-based decision making is a critical cornerstone of public policy. With the rapidly extending knowledge base on health and climate change, assessments to inform public policy are increasingly time and resource intensive. For example, the IPCC AR6 is expected in 2021 and 2022, almost 8 years since AR5 was published.

We have the knowledge, skills and techniques to perform comprehensive synthesis on a rolling basis—especially when computer assisted screening methodologies are applied alongside human screening. Such state-of-the-art synthesis of evidence will be crucial for development, implementation and decision-making around climate change adaptation responses and further stresses the importance of designing, conducting and clear reporting of climate change adaptation evaluations.

Information and knowledge on health adaptation can be found in traditionally difficult-to-access sources, including grey literature, policy and project evaluations, (archived) websites, and social media platforms. Extending the knowledge base on health adaptation can make use of advancements in digital methods and tools; web scraping can extend the scope of literature included; (pre-)trained algorithms can help predict relevant information sources, and advancements in automated translations could significantly decrease English reporting bias [34].

### 4.5. Strengths and limitations

We present a comprehensive systematic review and synthesis of published evidence on the effects on health of climate change adaptation responses—focussing on LMICs. The collated evidence provides important insights into the available published literature. The database of studies—on which analyses are based—is available in the supplementary material and forms a timely and substantial contribution to current climate change adaptation literature. However, there are several important limitations that need to be considered when interpreting the findings.

First, the evidence base is relatively restricted both in scale and in quality. The limited scale of the evidence base, reduced our ability to synthesise findings across multiple studies, which in turn restricts direct use of data for evidence-based decision making. The limited quality of the evidence base, with very few studies being graded as study design category 1 (i.e. *ex ante* formal evaluations of a defined adaptation responses), means that confidence in the reliability and strength of the findings is reduced. Second, there are limitations in the way in which the studies were identified. The original GAMI project was not designed specifically to answer the question on the effects on human health of climate change adaptation responses. While screening of the GAMI database of 48 813 papers for relevant studies was systematic, it is possible that some relevant papers were not included in the original GAMI database. It could be important to explore whether relevant climate-health literature is being indexed under very specific terms not captured by GAMI search terms (i.e. studies not using ‘adaptation’ or ‘risk’ in their abstracts and titles). Other biases, including those relating to reporting, publication and language, are common concerns in reviews of this nature and should be considered. Information on costs as well as barriers and enablers of adaptation options was not extracted from the included studies, as this was beyond the scope of this study. Given the data paucity on specific adaptation option such data would, however, give merely anecdotal evidence and costs could vary greatly from country to country. Finally, the review focuses on the currently available (and limited) scientific literature, meaning it does not include grey literature sources, nor does it provide much information on newly emergent areas of research. Recent areas of particular research activity include for example a focus on antimicrobial resistance and climate change, as well as a rapidly emerging literature on post-COVID19 climate governance. An update to this review in 3–5 years would certainly reveal several new priority areas.

## 5. Conclusions

There is an urgent need for significantly greater consideration of the health outcomes in evaluations of climate change adaptation responses, including pre-specified and well-designed formal evaluations. Pivotal for swift improvement on the evidence base will be specification of standardised approaches and reporting requirements of evaluations of effects on health of adaptation responses. Furthermore, further understanding of the effect of adaptation responses on all-cause mortality and cause-specific health outcomes, especially related to vulnerable groups including women, trans and non-binary people and children, will be crucial for evidence-based policy-making.

## Data Availability

All data that support the findings of this study are included within the article (and any supplementary files).

## References

[1] Pachauri R K, Allen M R, Barros V R, Broome J, Cramer W, Christ R, Church J A, Clarke L, Dahe Q, Dasgupta P (2014). Climate change 2014: synthesis report *Contribution of working groups i, ii and iii to the 5th Assessment Report of the Intergovernmental Panel on Climate Change: IPCC*. https://www.ipcc.ch/site/assets/uploads/2018/05/SYR_AR5_FINAL_full_wcover.pdf.

[2] Watts N, Amann M, Arnell N, Ayeb-Karlsson S, Belesova K, Boykoff M, Byass P, Cai W, Campbell-Lendrum D, Capstick S (2019). The 2019 report of the lancet countdown on health and climate change: ensuring that the health of a child born today is not defined by a changing climate. Lancet.

[3] Masson-Delmotte V, Zhai P, Pörtner H-O, Roberts D, Skea J, Shukla P R, Pirani A, Moufouma-Okia W, Péan C, Pidcock R (2018). Global warming of 1.5 °C. An IPCC Special Report on the Impacts of Global Warming of 1.5 °C.

[4] van Ruijven B J (2014). Enhancing the relevance of shared socioeconomic pathways for climate change impacts, adaptation and vulnerability research. Clim. Change.

[5] Haines A, Kovats R S, Campbell-Lendrum D, Corvalán C (2006). Climate change and human health: impacts, vulnerability and public health. Public Health.

[6] Byers E, Gidden M, Leclère D, Balkovic J, Burek P, Ebi K, Greve P, Grey D, Havlik P, Hillers A (2018). Global exposure and vulnerability to multi-sector development and climate change hotspots. Environ. Res. Lett..

[7] Herlihy N, Bar-Hen A, Verner G, Fischer H, Sauerborn R, Depoux A, Flahault A, Schütte S (2016). Climate change and human health: what are the research trends? A scoping review protocol. BMJ Open.

[8] Mertz O, Halsnæs K, Olesen J E, Rasmussen K (2009). Adaptation to climate change in developing countries. Environ. Manage..

[9] de Coninck H, Revi A, Babiker M, Bertoldi P, Buckeridge M, Cartwright A, Dong W, Ford J, Fuss S, Hourcade J (2018). Chapter 4: strengthening and implementing the global response. Global Warming of 1.5 °C.

[10] Holzkämper A (2017). Adapting agricultural production systems to climate change—what’s the use of models?. Agriculture.

[11] Challinor A J, Müller C, Asseng S, Deva C, Nicklin K J, Wallach D, Vanuytrecht E, Whitfield S, Ramirez-Villegas J, Koehler A-K (2018). Improving the use of crop models for risk assessment and climate change adaptation. Agric. Syst..

[12] Schauberger G, Hennig-Pauka I, Zollitsch W, Hörtenhuber S J, Baumgartner J, Niebuhr K, Piringer M, Knauder W, Anders I, Andre K (2020). Efficacy of adaptation measures to alleviate heat stress in confined livestock buildings in temperate climate zones. Biosyst. Eng..

[13] Conejos S, Langston C, Smith J (2014). Designing for better building adaptability: a comparison of adaptSTAR and ARP models. Habitat Int..

[14] Beckage B, Gross L J, Lacasse K, Carr E, Metcalf S S, Winter J M, Howe P D, Fefferman N, Franck T, Zia A (2018). Linking models of human behaviour and climate alters projected climate change. Nat. Clim. Change.

[15] Berrang-Ford L, Biesbroek R, Ford J D, Lesnikowski A, Tanabe A, Wang F M, Chen C, Hsu A, Hellmann J J, Pringle P (2019). Tracking global climate change adaptation among governments. Nat. Clim. Change.

[16] Ford J D, Berrang-Ford L (2016). The 4Cs of adaptation tracking: consistency, comparability, comprehensiveness, coherency. Mitigation Adapt. Strateg. Glob. Change.

[17] Tompkins E L, Vincent K, Nicholls R J, Suckall N (2018). Documenting the state of adaptation for the global stocktake of the Paris agreement. Wiley Interdiscip Rev. Clim. Change.

[18] Biesbroek R, Berrang‐Ford L, Ford J D, Tanabe A, Austin S E, Lesnikowski A (2018). Data, concepts and methods for large‐n comparative climate change adaptation policy research: a systematic literature review. Wiley Interdiscip. Rev. Clim. Change.

[19] Ebi K L, Boyer C, Bowen K J, Frumkin H, Hess J (2018). Monitoring and evaluation indicators for climate change-related health impacts, risks, adaptation, and resilience. Int. J. Environ. Res. Public Health.

[20] Austin S E, Biesbroek R, Berrang-Ford L, Ford J D, Parker S, Fleury M D (2016). Public health adaptation to climate change in OECD countries. Int. J. Environ. Res. Public Health.

[21] Lesnikowski A, Ford J, Berrang-Ford L, Paterson J, Barrera M, Heymann S (2011). Adapting to health impacts of climate change: a study of UNFCCC annex I parties. Environ. Res. Lett..

[22] Panic M, Ford J D (2013). A review of national-level adaptation planning with regards to the risks posed by climate change on infectious diseases in 14 OECD nations. Int. J. Environ. Res. Public Health.

[23] (EASAC) EASAC (2019). The imperative of climate action to protect human health in Europe. EASAC Policy Report 38.

[24] Austin S E, Ford J D, Berrang-Ford L, Araos M, Parker S, Fleury M D (2015). Public health adaptation to climate change in Canadian jurisdictions. Int. J. Environ. Res. Public Health.

[25] Moher D, Liberati A, Tetzlaff J, Dg A, Group P (2009). Preferred reporting items for systematic reviews and meta-analyses: the PRISMA statement. PLoS Med..

[26] Haddaway N R, Macura B, Whaley P, Pullin A S (2018). ROSES RepOrting standards for Systematic Evidence Syntheses: *pro forma*, flow-diagram and descriptive summary of the plan and conduct of environmental systematic reviews and systematic maps. Environ. Evid..

[27] Global adaptation mapping initiative. https://globaladaptation.github.io/.

[28] Berrang-Ford L, Lesnikowski A, Fischer A P, Siders A, Mach K J, Thomas A, Callaghan M, Haddaway N, Kerr R B, Biesbroek R (2021). The global adaptation mapping initiative (GAMI): Part 1—Introduction and overview of methods. https://research.utwente.nl/en/publications/the-global-adaptation-mapping-initiative-gami-part-1-introduction.

[29] Fischer A P, Callaghan M, Berrang-Ford L, Nielsen M, Sotnik G, Canosa I V, Haddaway N, Biesbroek R, Harper S, Minx J (2021). The global adaptation mapping initiative (GAMI): Part 2—Screening protocol. https://research.utwente.nl/en/publications/the-global-adaptation-mapping-initiative-gami-part-2-screening-pr.

[30] Lesnikowski A, Berrang-Ford L, Siders A, Haddaway N, Biesbroek R, Harper S, Minx J, de Perez E C, Reckien D, New M (2021). The global adaptation mapping initiative (GAMI): Part 3—Coding protocol. https://protocolexchange.researchsquare.com/article/pex-1242/v1.

[31] (2021). World Bank Country and Lending Groups. https://datahelpdesk.worldbank.org/knowledgebase/articles/906519-world-bank-country-and-lending-groups.

[32] Magnan A, Schipper E, Burkett M, Bharwani S, Burton I, Eriksen S, Gemenne F, Schaar J, Ziervogel G (2016). Addressing the risk of maladaptation to climate change. Wiley Interdiscip. Rev. Clim. Change.

[33] Berry P, Enright P M, Shumake-Guillemot J, Villalobos Prats E, Campbell-Lendrum D (2018). Assessing health vulnerabilities and adaptation to climate change: a review of international progress. Int. J. Environ. Res. Public Health.

[34] Huntingford C, Jeffers E S, Bonsall M B, Christensen H M, Lees T, Yang H (2019). Machine learning and artificial intelligence to aid climate change research and preparedness. Environ. Res. Lett..

